# Microscopic susceptibility anisotropy imaging

**DOI:** 10.1002/mrm.28303

**Published:** 2020-05-07

**Authors:** Enrico Kaden, Noemi G. Gyori, S. Umesh Rudrapatna, Irina Y. Barskaya, Iulius Dragonu, Mark D. Does, Derek K. Jones, Chris A. Clark, Daniel C. Alexander

**Affiliations:** ^1^ Centre for Medical Image Computing University College London London UK; ^2^ Great Ormond Street Institute of Child Health University College London London UK; ^3^ Cardiff University Brain Research Imaging Centre Cardiff University Cardiff UK; ^4^ Institute of Imaging Science Vanderbilt University Nashville TN USA; ^5^ Siemens Healthcare Ltd Frimley UK; ^6^ School of Psychology Australian Catholic University Melbourne VIC Australia

**Keywords:** brain white matter, gradient‐echo MR imaging, microscopic frequency shift, orientational tissue heterogeneity, spherical mean technique (SMT)

## Abstract

**Purpose:**

The gradient‐echo MR signal in brain white matter depends on the orientation of the fibers with respect to the external magnetic field. To map microstructure‐specific magnetic susceptibility in orientationally heterogeneous material, it is thus imperative to regress out unwanted orientation effects.

**Methods:**

This work introduces a novel framework, referred to as microscopic susceptibility anisotropy imaging, that disentangles the 2 principal effects conflated in gradient‐echo measurements, (a) the susceptibility properties of tissue microenvironments, especially the myelin microstructure, and (b) the axon orientation distribution relative to the magnetic field. Specifically, we utilize information about the orientational tissue structure inferred from diffusion MRI data to factor out the $B_{0}$‐direction dependence of the frequency difference signal.

**Results:**

A human pilot study at 3 T demonstrates proxy maps of microscopic susceptibility anisotropy unconfounded by fiber crossings and orientation dispersion as well as magnetic field direction. The developed technique requires only a dual‐echo gradient‐echo scan acquired at 1 or 2 head orientations with respect to the magnetic field and a 2‐shell diffusion protocol achievable on standard scanners within practical scan times.

**Conclusions:**

The quantitative recovery of microscopic susceptibility features in the presence of orientational heterogeneity potentially improves the assessment of microstructural tissue integrity.

## 1 INTRODUCTION

Gradient‐echo magnetic resonance (MR) imaging allows the mapping of the magnetic susceptibility distribution in living tissue. The frequency of spin precession, measurable with a gradient‐echo experiment, is proportional to the local magnetic field induced when the sample is placed in an external magnetic field $B_{0}$ and depends on the susceptibility properties of the material under investigation. This signal mechanism can be exploited to assess the iron content of tissue^1^, ^2^, ^3^ and the degree of myelination^4^, ^5^, ^6^ in the brain. A seminal observation is that both the magnitude and phase of the gradient‐echo signal in white matter depend on the orientation of the examined sample with respect to the magnetic field.^7^, ^8^, ^9^ This macroscopic, that is, voxel scale, anisotropy has been attributed to the annular architecture of myelin sheath and its anisotropic susceptibility properties at the molecular level. The myelination of axons is a key marker of neural functioning and health, yet it has been challenging to quantify the intrinsic magnetic susceptibility of myelin sheath since the gradient‐echo signal conflates the direction‐dependent microscopic susceptibility of the myelin structure and the intravoxel orientation distribution of the axons. Current techniques, however, do not separate these 2 contributing effects and thus lack specificity to microscopic susceptibility features.

In addition, bulk susceptibility variation may give rise to long‐range effects in the gradient‐echo scan that do not only affect the voxel under consideration, but also distant voxels. To detect microscopic field perturbations originating from tissue microstructure, we need to eliminate any nonlocal signal effects. This may be achieved by quantitative susceptibility mapping, which recovers the bulk distribution of scalar‐^10^, ^11^, ^12^ and tensor‐valued^13^ magnetic susceptibility from the gradient‐echo phase signal. Note, however, that these dipole‐field models alone cannot fully explain the frequency shift measurements in white matter tissue due to a local microstructural contribution to the gradient‐echo signal.^14^, ^15^ Alternatively, the frequency at a reference echo time, which is typically chosen short, may be subtracted from the signal, removing time‐invariant frequency components and thus the long‐range field inhomogeneity effects to a large degree.^16^, ^17^, ^18^ This frequency difference mapping (FDM) technique focuses on the time evolution of the gradient‐echo frequency. Either way, the residual signal depends on both the microscopic susceptibility structure and the axon orientations with respect to the magnetic field, which in practice may lead to ambiguities in the assessment of myelin pathology. For this reason, there is an unmet need to recover microscopic susceptibility features in the presence of fiber crossings and orientation dispersion, which are ubiquitous in the brain.^19^

In this work we propose a new gradient‐echo MRI framework, referred to as microscopic susceptibility anisotropy imaging, that factors out the confounding effects of the axon orientation distribution and provides proxy maps of microscopic susceptibility anisotropy irrespective of orientational tissue heterogeneity and magnetic field direction. More specifically, the microscopic frequency shift characterizes the frequency variation between parallel and perpendicular orientation of individual microenvironments relative to the external magnetic field. To disentangle the 2 principal contributors to the voxel‐scale anisotropy observed in the gradient‐echo signal, that is, microscopic susceptibility anisotropy and fiber orientation distribution, we utilize information about the orientational tissue structure inferred from diffusion MRI measurements using the spherical mean technique (SMT), a recently introduced method^20^, ^21^ for microscopic diffusion anisotropy mapping. The long‐range bulk field perturbations are eliminated with FDM. This proof‐of‐concept study demonstrates the first microscopic frequency shift maps at 3 T in humans, which in the brain are putatively sensitive to the myelination of axons, following previous work^17^, ^22^, ^23^, ^24^ that linked the frequency difference signal primarily to myelin sheath. The novel technique is feasible on clinical scanners, as it requires, in addition to a 2 *b*‐shell diffusion scan, only a dual‐echo gradient‐echo sequence acquired at 1 or 2 head orientations with respect to the magnetic field.

## 2 METHODS

### 2.1 Microdomain population model

The gradient‐echo MR signal is produced by a large population of tissue microenvironments, also referred to as microdomains, for example, (myelinated) axon segments in white matter, which have a distribution of orientations. In this work we adopt a phenomenological approach based on the FDM technique^17^, ^18^ and describe, at absolute echo time *t*, the effective frequency shift of a single microdomain as

(1) $$\delta \omega_{t_{0}}\left(\theta ,\;t\right)=\omega_{A,t_{0}}\left(t\right)\sin^{2}\left(\theta \right)$$

relative to a reference time $t_{0}$,^15^, ^25^ which is typically chosen short. The frequency shift depends on the angle $\theta \;=\;\text{arccos}(\left\langle {\hat{B}}_{0},\;u\right\rangle )$ between the direction ${\hat{B}}_{0}\;=\;B_{0}/\left\|B_{0}\right\|\in S^{2}$ of the external magnetic field and the orientation $u\in S^{2}$ of the microdomain, where $S^{2}\;=\;\{x\in R^{3}:\|x\|\;=\;1\}$ denotes the 2‐dimensional unit sphere. $\omega_{A,t_{0}}\left(t\right)$ quantifies the microscopic frequency shift at time *t* with respect to $t_{0}$ when a microscopic axon segment is oriented perpendicular to the magnetic field direction and hence is a marker of microscopic susceptibility anisotropy. This signal representation follows experimental observations^15^, ^17^, ^25^ on the time evolution and $B_{0}$‐direction dependence of the gradient‐echo frequency in white matter regions predominantly with a simple orientational architecture, that is, with moderate orientation dispersion and no fiber crossings. The frequency shift is commonly attributed to the compartmentalization of water pools in nervous tissue and the anisotropic susceptibility structure of myelin sheath.

The frequency shift *δω* of a single microdomain given in Equation (1) generates an extra component in the microscopic gradient‐echo signal

(2) $$\delta h_{t_{0}}\left(\theta ,\;t\right)=\exp\left(i\omega_{A,t_{0}}\left(t\right)\sin^{2}\left(\theta \right)\;\left[t-t_{0}\right]\right),$$

where *i* denotes the imaginary unit. This microscopic signal component is anisotropic and therefore depends on the axon orientation and magnetic field direction. The observable MR signal on the voxel scale comes from a large ensemble of microdomains that may feature fiber crossings, orientation dispersion, and undulation patterns. In particular, the axons typically do not run parallel to each other along the same direction.^26^, ^27^ The intravoxel orientational heterogeneity is described by an orientation distribution *p*(*u*), $u\in S^{2}$, which is non‐negative, that is, *p*(*u*) ≥ 0, antipodally symmetric, that is, *p*(*u*) = *p*(−*u*), and normalized to unity, that is, $\int_{S^{2}}p\left(u\right)\;du\;=\;1$. The spherical convolution of *p* with the microscopic signal shift *δh* from Equation (2), after replacing $\sin^{2}\left(\theta \right)\;=\;1-{\left\langle {\hat{B}}_{0},\;u\right\rangle }^{2}$, yields the macroscopic signal shift

(3) $$\delta E_{t_{0}}\left({\hat{B}}_{0},\;t\right)=\int_{S^{2}}\exp\left(i\omega_{A,t_{0}}\left(t\right)\;\left[1-{\left\langle {\hat{B}}_{0},\;u\right\rangle }^{2}\right]\;\left[t-t_{0}\right]\right)p\left(u\right)\;du$$

observed in gradient‐echo measurements. Note that this microdomain population model may also be formulated using a modern measure‐theoretic approach.^28^ Subsequently, the microscopic frequency shift $\omega_{A,t_{0}}\left(t\right)$ can be estimated based on Equation (3) once we have knowledge of the axon orientation distribution *p*, which may be obtained from diffusion MR imaging.

### 2.2 Experiment design

**Figure 1:**
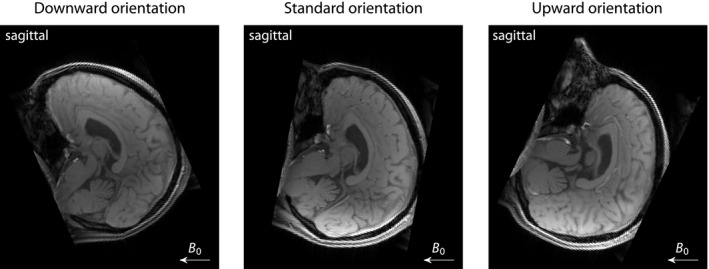
The gradient‐echo measurements were carried out at 3 different head orientations (downwards, standard, upwards) with respect to the external magnetic field. The rotation axis of the head inside the MRI scanner was left‐right; the angle between upward and downward head position was $59.8^{\circ }$ in this instance

To demonstrate microscopic susceptibility anisotropy imaging, we conducted a human pilot study with 3 healthy adult volunteers (2 24‐year‐old females, 1 male aged 27 years) after written informed consent had been obtained. The multi‐modal dataset was acquired on a 3 T Siemens Prisma system. Using a 20‐channel phased‐array head coil, a 3D flow‐compensated gradient‐echo sequence with bipolar signal readout measured a train of 11 gradient echoes with first echo time of 4.5 ms and inter‐echo spacing of 4.5 ms at 3 different head orientations (Figure 1). Note that the minimum requirements are 2 gradient echoes and a single head position. The flip angle was $21^{\circ }$ (Ernst angle) and the repetition time 53 ms. The gradient‐echo sequence employed GRAPPA parallel imaging with an acceleration factor of 2. The measurement of the 128 × 160 × 96 image matrix with field of view of $192\;\times \;240\;\times \;144\;{\mathrm{mm}}^{3}$, which was adjusted for each head rotation, covered the whole brain, resulting in an isotropic voxel resolution of 1.5 mm. The per‐channel magnitude and phase image data were reconstructed using the product *k*‐space data processing pipeline. The acquisition time was circa 8 minutes per head position.

In addition, a Stejskal–Tanner EPI sequence measured 2 *b*‐shells of nominally 1000 and $2500\;s/{\text{mm}}^{2}$ with 60 evenly distributed gradient directions each^29^ using a 64‐channel phased‐array head coil, while keeping the gradient timing fixed during the experiment. 10 images without diffusion weighting were collected. The scan with echo time of 72.8 ms and repetition time of 3.8 seconds was acquired twice with the signal readout direction reversed, using a multiband sequence^30^, ^31^ with a slice acceleration factor of 3 and GRAPPA parallel imaging with an in‐plane acceleration factor of 2. SENSE1 multiple‐coil combination^32^, ^33^ was applied. The measurement of 96 slices with 1.5 mm thickness, 154 × 154 image matrix and field of view of $230\;\times \;230\;{\mathrm{mm}}^{2}$ yielded a voxel resolution of $1.49\;\times \;1.49\;\times \;1.5\;{\mathrm{mm}}^{3}$. The scan time was approximately 18 minutes. For anatomical reference, we acquired a 3D $T_{1}$‐weighted MP‐RAGE volume at isotropic resolution of 1 mm. The study was approved by the Joint Research Ethics committee of the NHS Great Ormond Street Hospital for Children and UCL Great Ormond Street Institute of Child Health.

### 2.3 Frequency difference mapping

This work uses gradient‐echo measurements only from the odd‐numbered echoes of the echo train to avoid potential adverse effects in the statistical analysis due to differences in the signal formation between odd‐ and even‐numbered echoes. First, we align the $T_{1}$‐weighted MR images of all study participants to the axes of MNI152 space through rigid‐body transformations without scaling, maintaining the subjects’ brain size and shape.^34^, ^35^ The brain is masked in the MP‐RAGE volumes^36^ and gross anatomical structures are segmented with FreeSurfer.^37^, ^38^ Next, after multiple‐coil combination of the per‐channel magnitude and phase image data,^39^ motion correction,^40^ and Gibbs ringing suppression with a Hann window filter, the gradient‐echo MR images from the different head positions are co‐registered with the $T_{1}$‐weighted scan in (unscaled) MNI152 space using affine transformations,^34^ in order to reduce residual distortions, while adjusting the magnetic field direction accordingly. Following phase unwrapping,^41^ the frequency shift at echo time *t* is computed with respect to the first gradient echo at $t_{0}\;=\;4.5\;\mathrm{ms}$. The FDM technique^17^, ^18^ eliminates time‐independent frequency components and thus the long‐range field inhomogeneity effects to a large extent. Subsequently, we estimate residual large‐scale frequency variation, such as the initial phase offset, using second‐order 3D total generalized variation regularization^42^ and remove it from the frequency shift signal for each echo time *t* and magnetic field direction ${\hat{B}}_{0}$ separately (see Supporting Information Text). This step factors out spatially slowly varying phase contributions up to a global additive constant $\phi_{{\hat{B}}_{0},t}$, which depends on the total image content.

### 2.4 Spherical mean technique

The information about the orientational heterogeneity is obtained from the diffusion images, which we preprocess first. Briefly, after Gibbs ringing suppression,^43^ susceptibility‐induced distortions, eddy‐current artifacts, and subject motion are corrected using information gained from the reversed phase‐encoding polarity.^44^ The diffusion‐weighted volume is then co‐registered to the MP‐RAGE scan in (unscaled) MNI152 space through rigid‐body transformations. Since the MR signal has been combined with SENSE1 from multiple receive coils, the noise regime of the magnitude signal is well described by a Rician distribution, albeit data preprocessing may alter its characteristics to a certain extent. To minimize potential side effects of the noise‐induced bias, the measurements are adjusted accordingly.^21^

To recover the axon orientation distribution quantitatively, we use SMT, a recently introduced method for microscopic diffusion anisotropy imaging^20^, ^21^ that maps the microscopic diffusion process unconfounded by and without knowledge of the orientational tissue architecture including fiber crossings and orientation dispersion. Two microscopic models, which describe the microscopic diffusion signal from a single microdomain, are fitted to the data, that is, a microscopic diffusion tensor,^21^ which is a second‐order approximation of the microscopic diffusion process, and a microscopic compartment model,^20^ which provides an estimate of the intra‐axonal volume fraction. Once the microscopic diffusion signal has been determined voxel by voxel, we estimate the axon orientation distribution *p* using spherical deconvolution, which here, unlike traditional methods,^45^, ^46^ utilizes a spatially varying impulse response function. The orientation distribution function is represented in real‐valued spherical harmonic basis with maximum order of 8 and constrained to be non‐negative, antipodally symmetric, and normalized to unity.

### 2.5 Microscopic susceptibility anisotropy mapping

Subsequently, we estimate the microscopic frequency shift $\omega_{A,t_{0}}\left(t\right)$ from the gradient‐echo frequency signal at echo time *t* and the axon orientation distribution *p*. The biophysical model formulated in Equation (3) may be numerically approximated by

(4) $$\delta E_{t_{0}}\left({\hat{B}}_{0},\;t\right)=\frac{1}{N}\sum_{j=1}^{N}\exp\left(i\omega_{A,t_{0}}\left(t\right)\left[1-{\left\langle {\hat{B}}_{0},\;u_{j}\right\rangle }^{2}\right]\left[t-t_{0}\right]\right)p\left(u_{j}\right),$$

where $u_{j}\in S^{2}$ denote the *N* = 500 orientation samples which together with $-u_{j}$ are evenly distributed on the sphere. It is sufficient to sample only a hemisphere since *p* is antipodally symmetric. Alternatively, the microdomain population model (3) may be represented in spherical harmonic basis to compute convolutions of functions more efficiently (see Appendix A).

To recover the microscopic frequency shift ${\tilde{\omega }}_{A,t_{0}}\left(t\right)$ unconfounded by the axon orientation distribution, we solve voxel by voxel the optimization problem

(5) $${\tilde{\omega }}_{A,t_{0}}\left(t\right)=\arg\min_{\omega_{A,t_{0}}\in \Omega }\;\sum_{{\hat{B}}_{0}}D{\left\{\exp\left(iy_{t_{0}}\left({\hat{B}}_{0},t\right)\left[t-t_{0}\right]\right),\text{sgn}\left(\delta E_{t_{0}}\left({\hat{B}}_{0},t\right)\right)\right\}}^{2}$$

over a specified frequency interval Ω for a set of frequency shift measurements $y_{t_{0}}\left({\hat{B}}_{0},\;t\right)$ taken at magnetic field direction ${\hat{B}}_{0}$ and echo time *t*. The error metric reads $D\left\{z_{1},\;z_{2}\right\}\;=\;\text{arccos}\left(\text{Re}\left(z_{1}^{*}z_{2}\right)\right)$ and computes the phase difference between $z_{1}$ and $z_{2}$ with $|z_{i}|\;=\;1$, where the asterisk denotes the complex conjugate and Re(*z*) the real part of a complex number *z*. In general, a nontrivial microdomain orientation distribution may not only produce a macroscopic phase shift, but also a decay of the gradient‐echo signal magnitude, which here is not exploited further. We focus on the phase content using the signum function sgn(*z*) = *z*/|*z*| defined for complex numbers *z* except zero. The estimation problem (5) is nonlinear and may have multiple solutions due to phase wrapping. Therefore, we adopt a simple heuristic, assuming that the microscopic frequency shift evolves smoothly in time, and perform model fitting, at echo time $t_{k+1}$, over the frequency interval $\Omega_{k+1}\;=\;\left[{\tilde{\omega }}_{A,t_{0}}\left(t_{k}\right)-\pi /t_{k+1},\;{\tilde{\omega }}_{A,t_{0}}\left(t_{k}\right)+\pi /t_{k+1}\right]$ that is iteratively adjusted with respect to the frequency shift ${\tilde{\omega }}_{A,t_{0}}\left(t_{k}\right)$ estimated at $t_{k}$. If there is no information available from a preceding gradient echo, the search interval is centred at 0.

As discussed in Section 2.3, the frequency shift measurement $y_{t_{0}}\left({\hat{B}}_{0},\;t\right)$ may be known only up to an arbitrary frequency offset $\phi_{{\hat{B}}_{0},t}$, which remains undetermined after the removal of residual large‐scale frequency variation, is spatially constant and generally varies with magnetic field direction ${\hat{B}}_{0}$ and echo time *t*. If $\phi_{{\hat{B}}_{0},t}$ cannot be obtained in a different way, we estimate the global additive constants for each *t* and ${\hat{B}}_{0}$ jointly with the microscopic frequency shift ${\tilde{\omega }}_{A,t_{0}}\left(t\right)$ as follows. For a set of frequency offsets $\left\{\phi_{{\hat{B}}_{0},t}\right\}$, the microscopic frequency shift is fitted voxel by voxel, using Equation (5), to the frequency difference signals with the current frequency offsets applied. The unknown frequency constants $\left\{\phi_{{\hat{B}}_{0},t}\right\}$ are then iteratively optimized with respect to the total estimation error over a region of interest, for example, brain tissue. Note that this method for determining $\left\{\phi_{{\hat{B}}_{0},t}\right\}$ requires at least 2 head orientations with respect to the magnetic field.

### 2.6 Noise amplification

The microscopic axon segments that are oriented parallel to the main magnetic field do not contribute to the observable macroscopic frequency shift, irrespective of their degree of myelination and microscopic susceptibility anisotropy. Nevertheless, as we will show in Section 3, it is almost always possible to recover the microscopic frequency shift since the intravoxel orientational architecture is typically heterogeneous on the millimeter scale, even in the densest and most coherent white matter regions like the corpus callosum or internal capsule.^26^, ^27^ However, we may observe a noise amplification effect depending on the microdomain orientation distribution and the acquired set of magnetic field directions. The model fitting problem as formulated in Equation (5) is nonlinear, thus we linearize the functional relationship around the maximum likelihood solution ${\tilde{\omega }}_{A,t_{0}}\left(t\right)$ to simplify the uncertainty quantification. The noise amplification factor *g* may be approximated in terms of

(6) $$g=\sqrt{{\left(J_{{\tilde{\omega }}_{A,t_{0}}\left(t\right)}^{T}J_{{\tilde{\omega }}_{A,t_{0}}\left(t\right)}^{\;}\right)}^{-1}},$$

where

(7) $$J_{{\tilde{\omega }}_{A,t_{0}}\left(t\right)}={\left[\frac{D\{\text{sgn}\left(\delta E_{t_{0}}\left({\tilde{\omega }}_{A,t_{0}}\left(t\right)+\epsilon ;\;{\hat{B}}_{0},t\right)\right),\;\text{sgn}\left(\delta E_{t_{0}}\left({\tilde{\omega }}_{A,t_{0}}\left(t\right)-\epsilon ;\;{\hat{B}}_{0},\;t\right)\right)\}}{2\epsilon }\right]}_{{\hat{B}}_{0}}$$

denotes the *n* × 1‐dimensional gradient vector with the central‐difference numerical derivatives at ${\tilde{\omega }}_{A,t_{0}}\left(t\right)$ with respect to *n* magnetic field directions ${\hat{B}}_{0}$ for a sufficiently small *ϵ* > 0. We assume that Gaussian noise in the gradient‐echo frequency measurements is uncorrelated and has the same level across ${\hat{B}}_{0}$. The *g*‐factor may then be calculated voxel by voxel.

## 3 RESULTS

**Figure 2:**
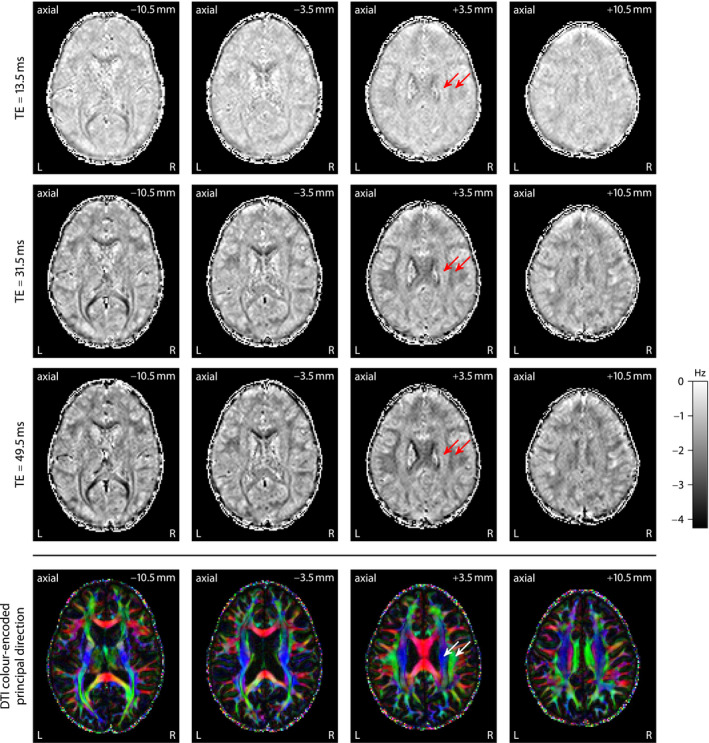
Frequency difference mapping,^17^, ^18^ shown for 3 echo times at a single magnetic field direction in various axial planes, demonstrates that the gradient‐echo frequency shift contrast is largely due to its dependence on echo time and the orientational heterogeneity of brain white matter. For comparison, the bottom row depicts the DTI color‐encoded principal direction. Left (L), right (R)

It has been shown that the gradient‐echo frequency in brain white matter depends on the echo time and the axon orientations with respect to the external magnetic field.^7^, ^8^, ^9^ Figure 2 replicates these previous findings, which are demonstrated for various echo times (from top to bottom) at a single magnetic field direction in 4 axial planes (from left to right) using FDM with respect to the first gradient echo at 4.5 ms.^17^, ^18^ The frequency difference signal has factored out time‐invariant frequency components as well as long‐range field inhomogeneity effects to a large extent. The 2 white matter regions marked with arrows point to fiber bundles, that is, the pyramidal tract and the superior longitudinal fasciculus, which mostly run parallel and perpendicular to the magnetic field in standard head position, respectively. For reference, the bottom row displays the color‐encoded principal direction obtained from diffusion tensor imaging (DTI).^47^ Figure 2 demonstrates a striking contrast in the macroscopic frequency shift between these 2 regions, which is primarily attributed to the $B_{0}$‐direction dependence of the gradient‐echo measurement. This effect makes it cumbersome to quantify microscopic magnetic susceptibility or proxies thereof from only a single gradient‐echo scan.

**Figure 3:**
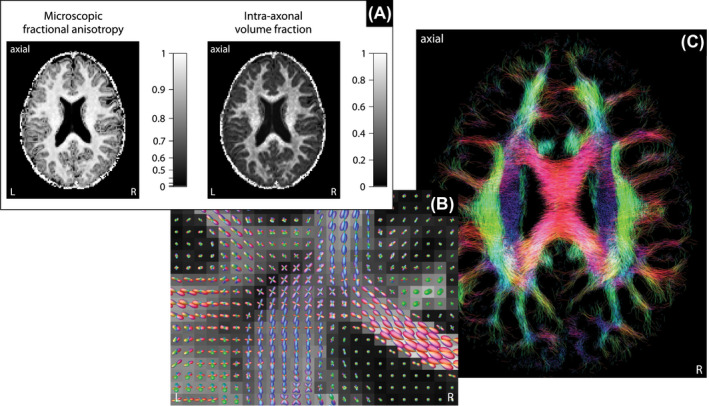
Spherical Mean Technique (SMT)^20^, ^21^ for microscopic diffusion anisotropy mapping. (a) Estimation of microscopic diffusion features, such as microscopic fractional anisotropy and intra‐axonal volume fraction, unconfounded by fiber crossings and orientation dispersion. (b) Quantitative recovery of the axon orientation distribution using spherical deconvolution with a spatially varying impulse response function, showing the crossing of the callosal fibers and the pyramidal tract in the centrum semiovale. (c) Probabilistic tractography,^48^ visualized with MRtrix3, revealing the complex architecture of the fiber pathways in the individual human brain

We take advantage of SMT‐based microscopic diffusion anisotropy imaging^20^, ^21^ to estimate the axon orientation distribution, which is subsequently used to map microscopic susceptibility anisotropy. Figure 3 gives an overview of the recently proposed technique, which disentangles microscopic diffusion features from fiber crossings and orientation dispersion using a widely available off‐the‐shelf diffusion sequence. Panel A maps the microscopic fractional anisotropy, obtained from a microscopic tensor model,^21^ and the intra‐axonal volume fraction, estimated from a clinically viable microscopic compartment model.^20^ The voxel‐by‐voxel microscopic diffusion signal informs the spatially varying impulse response function that is used to recover, in a second step, the axon orientation distribution through spherical deconvolution, as shown in Panel B of the figure, here exposing the orientational heterogeneity in the centrum semiovale. The underlying map depicts the DTI fractional anisotropy. Afterwards, we are able to perform fiber tractography^48^ and quantify anatomical connectivity.^49^ It is evident from Panel C that diverging, converging, and crossing pathways are ubiquitous in brain white matter, which give rise to the high prevalence of complex orientation distributions at the millimeter resolution of the MRI measurement.

**Figure 4:**
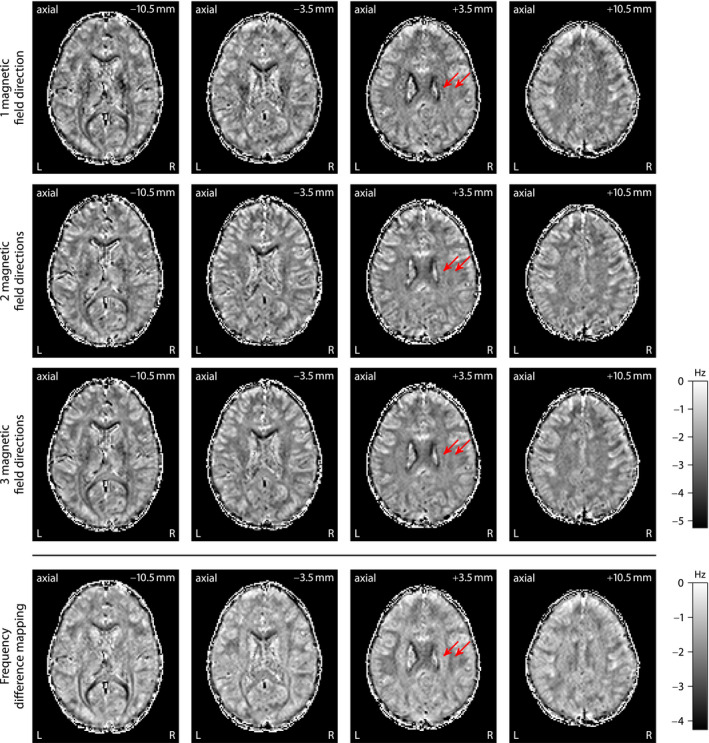
Maps of the microscopic frequency shift $\omega_{A,t_{0}}\left(t\right)/\left(2\pi \right)$ obtained from gradient‐echo measurements with different numbers of magnetic field directions at echo time *t* = 40.5 ms. For comparison, the fourth row shows the macroscopic frequency shift for a single $B_{0}$‐direction before factoring out orientational heterogeneity. The 2 arrows point to white matter regions with fiber bundles predominantly running parallel (left) and perpendicular to the external magnetic field in standard head orientation

Next, we estimate microscopic susceptibility anisotropy from the frequency difference signal (Figure 2) using the axon orientation information obtained with SMT‐based microscopic diffusion tensor mapping (Figure 3). Figure 4 plots maps of the microscopic frequency shift $\omega_{A,t_{0}}\left(t\right)/\left(2\pi \right)$ unconfounded by fiber crossings and orientation dispersion as well as magnetic field direction. The figure shows the results from gradient‐echo measurements with different numbers of $B_{0}$‐directions (from top to bottom) at echo time *t* = 40.5  ms with respect to the reference time $t_{0}\;=\;4.5\;\;\mathrm{ms}$. A central outcome is that the microscopic frequency shift, a marker of microscopic susceptibility structure, has less variability in brain white matter compared to the frequency difference signal. For example, the pyramidal tract (left arrow) and the superior longitudinal fasciculus (right arrow) have markedly different macroscopic frequency shifts as demonstrated in the bottom row of the figure, whereas this contrast disappears to a large extent in the microscopic susceptibility maps that have factored out the $B_{0}$‐direction dependence. An important feature is that the proposed technique requires only a few head orientations with respect to the magnetic field direction. We achieve reasonable results even with a single head position (top row of Figure 4) under the premise that the global frequency offset is known, which in this work has been obtained from a model fit with 3 magnetic field directions, but may be estimated differently.^17^, ^18^ See Supporting Information Figures S1‐S6 for more experimental results, including preliminary data using a 32‐channel phased‐array head coil that offers a higher signal‐to‐noise ratio than the 20‐channel coil mainly used in this study, at the expense of a lower $B_{0}$‐direction encoding capacity due to limited room for head movement (Supporting Information Figure S1).

**Figure 5:**
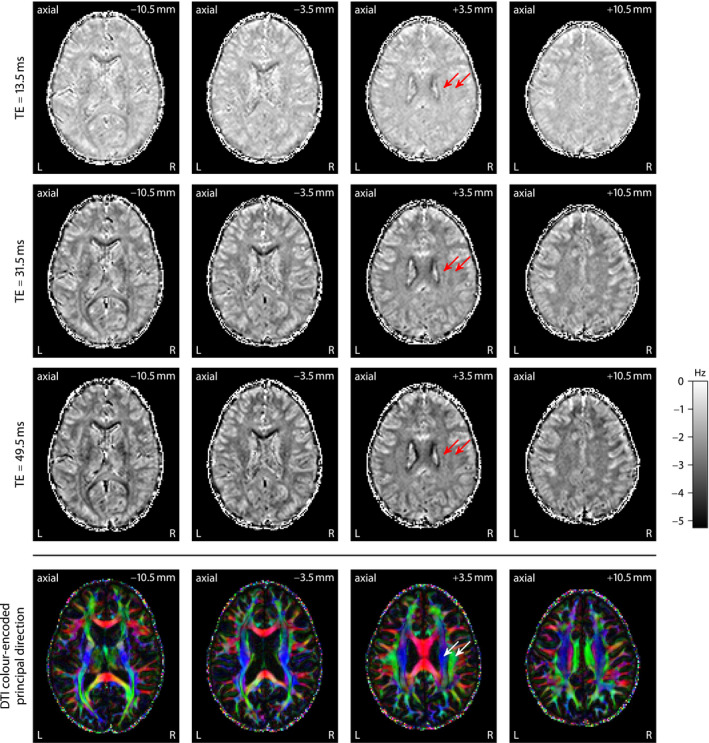
Time dependence of the microscopic frequency shift $\omega_{A,t_{0}}\left(t\right)/\left(2\pi \right)$, here estimated from 3 magnetic field directions at various echo times. The bottom section maps the DTI color‐encoded principal direction, showing the orientational heterogeneity in brain white matter. The 2 arrows indicate the pyramidal tract (left) and the superior longitudinal fasciculus, which are largely oriented parallel and perpendicular to the main magnetic field in standard head position, respectively

Figure 5 maps the time evolution of $\omega_{A,t_{0}}\left(t\right)/\left(2\pi \right)$, which gives the effective frequency shift at echo time *t* if a tissue microenvironment, here a microscopic axon segment, were oriented perpendicular to the external magnetic field. This microscopic frequency shift is estimated using Equation (5) from 3 magnetic field directions at different echo times (from top to bottom) with respect to the reference echo time of 4.5 ms. The spatial contrast in these maps comes primarily from the segregation of brain tissue into gray and white matter, which likely reflects the content of myelin. The microscopic frequency shift plots show much less contrast within white matter than between gray and white matter, unlike the frequency difference signal (Figure 2) that exhibits high contrast within white matter due to the $B_{0}$‐direction dependence. Moreover, the image contrast between gray and white matter in Figure 5 increases with longer echo time, that is, the microscopic frequency shift in brain white matter becomes more negative, whereas in gray matter the degree of anisotropy remains at a low level. This time dependence of the microscopic magnetic susceptibility index potentially conveys rich information about the microscopic structure of myelin sheath^17^ unconfounded by the axon orientation distribution. We obtain similar results for the other subjects (not shown).

**Figure 6:**
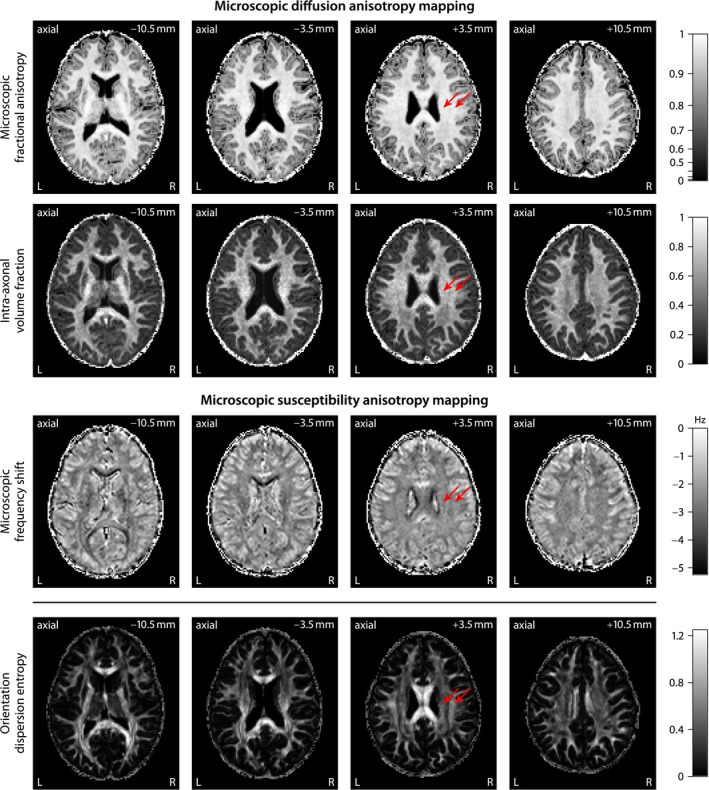
Microscopic anisotropy mapping both in diffusion and susceptibility MRI. An important observation is that all these microscopic feature maps are relatively homogeneous in brain white matter because the confounding effects due to fiber crossings and orientation dispersion as well as magnetic field and diffusion gradient direction have been factored out. For comparison, the bottom row shows the orientation dispersion entropy that quantifies the orientational tissue heterogeneity^20^

Figure 6 compares microscopic diffusion and susceptibility anisotropy imaging. The first and second row show the microscopic fractional anisotropy and intra‐axonal volume fraction,^20^, ^21^ while the third row maps the microscopic frequency shift at echo time *t* = 40.5  ms with respect to 4.5 ms. The latter is obtained from a gradient‐echo scan with 3 magnetic field directions and diffusion‐based information about the axon orientation distribution. These microscopic parameter maps suggest that the tissue microanatomy is relatively homogeneous within white matter and also within gray matter, in comparison to the marked differences between white and gray matter, regarding the microscopic diffusion and susceptibility features. In contrast, the bottom row of the figure maps a summary statistic of the microdomain orientation distribution, that is, the relative entropy with respect to the uniform distribution,^20^ showing the orientational heterogeneity of brain tissue. For instance, the pyramidal tract (left arrow) fans out over a wide area of the cerebral cortex, thus has a lower orientation dispersion entropy and runs mostly parallel to the external magnetic field in standard head position. The superior longitudinal fasciculus links cortical areas within the same hemisphere along the anterior‐posterior direction mainly perpendicular to $B_{0}$ and is relatively coherent in the region to which the right arrow points, thus has a higher orientation dispersion entropy. The microscopic, orientation‐invariant feature maps do not show the spatial contrast to the extent we observe in the raw diffusion and gradient‐echo signals, which are dominated by orientation effects.

**Figure 7:**
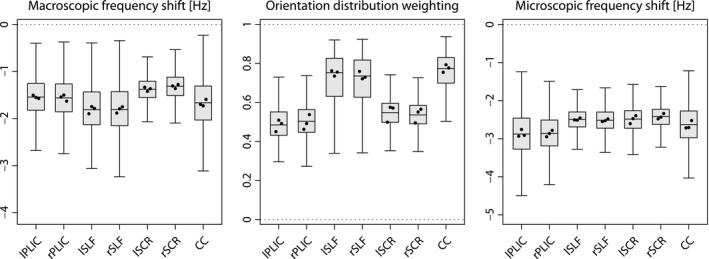
The box plots (with 1.5‐times the interquartile range) show, from left to right, the macroscopic frequency shift and orientation distribution weighting for the gradient‐echo measurement in standard head position as well as the microscopic frequency shift, after regressing out the effects of fiber crossings and orientation dispersion, in various white matter regions for 3 healthy adults. The dots depict the median of individual subjects. Posterior limb of internal capsule (PLIC), superior longitudinal fasciculus (SLF), superior corona radiata (SCR), corpus callosum (CC); prefix: left (l), right (r)

For a region‐based analysis, the brain white matter is automatically labeled using the ICBM‐DTI‐81 white‐matter atlas.^50^, ^51^ Figure 7 compares, in 3 healthy adult volunteers, the macroscopic frequency shift (left), which we observe for a single gradient‐echo measurement at echo time *t* = 40.5  ms with respect to the reference time of 4.5 ms, and the microscopic frequency shift (right), where the effects due to fiber crossings and orientation dispersion have been factored out. The middle box‐and‐whisker plot (with 1.5‐times the interquartile range) depicts the orientation distribution weighting $\pi ({\hat{B}}_{0})$ intrinsic to the gradient‐echo experiment as derived from Equation (3) in the short‐time limit, which takes the form

(8) $$\pi \left({\hat{B}}_{0}\right)=\int_{S^{2}}\left(1-{\left\langle {\hat{B}}_{0},\;u\right\rangle }^{2}\right)p\left(u\right)\;du$$

with ${\hat{B}}_{0}$ denoting the magnetic field direction and *p* the axon orientation distribution. $\pi ({\hat{B}}_{0})$ can be easily calculated using the spherical harmonic representation of *p* and lies in the range between 0 (ie, all microscopic axon segments are oriented parallel to ${\hat{B}}_{0}$) and 1 (ie, all segments are oriented perpendicular to ${\hat{B}}_{0}$). The fiber pathways in the posterior limb of the internal capsule and the superior corona radiata are mainly oriented parallel to the external magnetic field in standard head position, while the superior longitudinal fasciculus and the corpus callosum are largely oriented perpendicular to the magnetic field direction. Note that the variability in the plotted metrics (across regions and subjects) is partly due to the extent of the chosen regions of interest.

**Figure 8:**
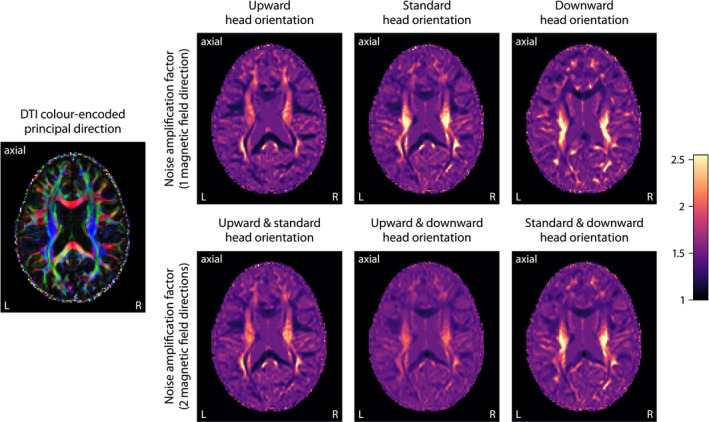
Noise amplification effect in microscopic susceptibility anisotropy imaging. The right panel maps the amplification factor for different sets of magnetic field directions at echo time of 40.5 ms. The acquisition of multiple head orientations with respect to the $B_{0}$‐direction (especially in upward and downward position) significantly reduces noise amplification in brain regions where otherwise the fiber pathways predominantly run parallel to the external magnetic field

Lastly, Figure 8 shows the noise propagation in microscopic susceptibility anisotropy imaging. The plots map the noise amplification factor *g* for different sets of head orientations relative to the external magnetic field at echo time of 40.5 ms, here with respect to plain averaging of the gradient‐echo frequency signal and thus corrected for differences in scan time. For a single $B_{0}$‐direction, we may observe a high degree of noise amplification in those regions where the fiber pathways mostly run parallel to the main magnetic field in the respective head position, noting that *g* is modulated by the orientation dispersion within the fiber bundle.^49^ The inverse problem is in general ill‐posed even with full knowledge of the axon orientation distribution. The figure also demonstrates that noise amplification can be greatly reduced with the measurement of 2 or more head orientations relative to the magnetic field direction. Especially for a gradient‐echo experiment at upward and downward head position (with an angle of $59.8^{\circ }$ in this instance), we achieve a significant reduction of *g*‐factor peaks since the acquisition of a second $B_{0}$‐direction contributes additional information on the microscopic axon segments that otherwise are oriented parallel to the external magnetic field, ultimately rendering the estimation problem well‐behaved. Even though we obtain reasonable results with a single magnetic field direction, it is favorable to acquire a gradient‐echo scan with at least 2 head orientations with respect to the $B_{0}$‐direction, in particular for quantitative mapping of microscopic susceptibility anisotropy.

## 4 DISCUSSION

In this work we have introduced an MRI technique that enables the in vivo recovery of the microscopic frequency shift, a marker of microscopic susceptibility anisotropy, in the presence of orientational tissue heterogeneity. The gradient‐echo signal does not only depend on the magnetic susceptibility properties of the tissue microenvironments, but also on their orientation distribution, which, at image resolution, is complex in the brain. Indeed, the neural circuitry is throughout characterized by fiber crossings and orientation dispersion^19^ and even white matter regions like the corpus callosum and internal capsule, which are typically considered coherent, feature a nontrivial orientational structure.^26^, ^27^ Therefore, to quantify microscopic magnetic susceptibility, it is imperative to regress out the unwanted effects of the axon orientation distribution and magnetic field direction. The orientation information is recovered from diffusion‐weighted measurements using SMT. This microscopic diffusion anisotropy imaging method^20^, ^21^ allows us, without prior knowledge of the orientational fiber architecture, to determine the per‐voxel impulse response function required for quantitative spherical deconvolution of the axon orientation distribution.

The microscopic frequency shift model is a descriptive representation of the local gradient‐echo signal contributions arising from the microscopic architecture of brain tissue, which cannot be explained by scalar‐ or tensor‐valued bulk susceptibility variation.^15^ The echo‐time and $B_{0}$‐direction dependence of the macroscopic, that is, voxel‐scale, frequency shift has been attributed to (a) the anisotropic susceptibility structure of myelin sheath, which is organized in annular layers, (b) the axon orientations relative to the external magnetic field, and (c) the compartmentalization of water pools with distinctly different transverse relaxation properties, that is, water trapped between the myelin bilayers and the water pools inside the axons and in the extra‐axonal space.^17^, ^22^, ^23^, ^24^ In this proof‐of‐concept study we have factored out the orientation effects in frequency difference measurements, making the microscopic frequency shift a putative marker of myelin microstructure unconfounded by fiber crossings and orientation dispersion (see Kaden et al.^52^ for initial validation). Indeed, the main contrast in the microscopic parameter maps is due to the segregation of brain tissue into white and gray matter. In comparison to the raw frequency difference signal, we observe much less variability across white matter tissue, which is in agreement with known myelin neuroanatomy.

The present work uses FDM to eliminate long‐range dipole effects, limiting us to studying the change of frequency in time. However, the general idea of the developed technique extends, after field‐to‐source inversion^10^, ^11^, ^13^ for removal of nonlocal bulk field perturbations, to absolute frequency measurements, which may contain additional information. The frequency difference signal is known only up to a global additive constant that typically depends on the image content and hence needs to be calibrated, for example, to the frequency signal of cerebrospinal fluid^17^ or gray matter,^18^ which, however, was not sufficiently reliable in our experimental setting. Therefore, we estimate the global frequency offset simultaneously with the microscopic frequency shift, which requires a gradient‐echo scan at 2 or more head orientations with respect to the external magnetic field. The microscopic susceptibility model we employ here is a phenomenological signal description that follows experimental results and computer simulations.^15^, ^17^, ^25^ Note, however, that signal subcomponents, for example, originating from isotropic susceptibility of myelin sheath or orientation‐independent chemical exchange, may deviate from the $B_{0}$‐direction dependence model in Equation (1), although these effects do not seem to dominate the frequency difference measurements in the living human brain.^17^ The microscopic frequency shift consistently shows negative contrast in white matter presumably because the $T_{2}^{*}$‐relaxation time of myelin water, whose average frequency shift is positive with respect to the intra‐ and extra‐axonal compartments,^17^, ^53^ is relatively short. General limitations are susceptibility‐induced distortions especially at longer echo times, residual spatial misalignment of gradient‐echo acquisitions at different head positions and image artifacts due to subject motion, which are common challenges in gradient‐echo imaging. The optimal choice of the head coil is a trade‐off between the number of receive channels and room for head movement, which control the signal‐to‐noise ratio and $B_{0}$‐direction encoding capacity, and needs to be determined for individual cohorts.

In conclusion, microscopic susceptibility anisotropy mapping is a new gradient‐echo MRI framework that imposes only modest experimental requirements on scanner hardware and the number of head orientations with respect to the magnetic field direction, yet offers excellent opportunities in brain research and clinical neurology. The proposed technique may be particularly useful for the in vivo assessment of myelin microstructure, ranging from myelin formation during brain development^54^ to myelin breakdown in various neurological conditions such as multiple sclerosis.^55^ A unique feature is that the developed imaging biomarkers are not confounded by the $B_{0}$‐direction dependence of the gradient‐echo signal. More advanced microscopic models, which, for instance, exploit the time evolution of frequency shift anisotropy,^17^, ^56^ provide a future avenue for the detailed investigation of magnetic susceptibility structure at the microscopic level in orientationally heterogeneous tissue. To improve practicability, we envisage a custom‐designed pillow system that facilitates the reliable measurement in upward and/or downward head position. Gradient‐echo sequences with EPI^57^ or Wave‐CAIPI readout^58^ may be used to reduce the scan time.

## CONFLICT OF INTEREST

Iulius Dragonu is an employee of Siemens Healthcare Ltd, Frimley, UK.

## Supporting information


**FIGURE S1** Maps of the microscopic frequency shift $\omega_{A,t_{0}}\left(t\right)/\left(2\pi \right)$, similar to Figure 4, obtained from gradient‐echo measurements with different numbers of magnetic field directions, but acquired with a 32‐channel phased‐array head coil offering a substantially higher signal‐to‐noise ratio at the expense of significantly less room for head movement that limits the achievable maximum angle between head orientations (here $39.2^{\circ }$)
**FIGURE S2** Maps of the microscopic frequency shift $\omega_{A,t_{0}}\left(t\right)/\left(2\pi \right)$ estimated from a single gradient‐echo scan for different head orientations at echo time *t* = 40.5 ms. For comparison, the bottom row shows $\omega_{A,t_{0}}\left(t\right)/\left(2\pi \right)$ obtained from all 3 head positions relative to the main magnetic field. The 2 arrows point to white matter regions with fiber bundles mostly running parallel (left) and perpendicular to the external magnetic field in standard head orientation
**FIGURE S3** Differences of the microscopic frequency shift estimates from single head orientations with respect to a gradient‐echo experiment comprising all 3 head positions relative to the external magnetic field (bottom row) at echo time of 40.5 ms. Note that head orientation specific noise amplification effects, susceptibility‐induced image distortions, and residual spatial misalignment contribute to the variation in microscopic susceptibility anisotropy mapping
**FIGURE S4** Microscopic frequency shift $\omega_{A,t_{0}}\left(t\right)/\left(2\pi \right)$ obtained from gradient‐echo measurements at 2 head positions relative to the external magnetic field at echo time of 40.5 ms. For comparison, the bottom row shows the microscopic susceptibility anisotropy index estimated from all 3 head orientations. The 2 arrows indicate the pyramidal tract (left) and the superior longitudinal fasciculus, which are primarily oriented parallel and perpendicular to the main magnetic field in standard head position, respectively
**FIGURE S5** Differences of the microscopic frequency shift estimates from 2 head orientations with respect to a gradient‐echo measurement comprising all 3 head positions relative to the external magnetic field (bottom row) at echo time *t* = 40.5 ms. Note that orientation‐dependent noise amplification, susceptibility‐induced distortions, and residual spatial misalignment contribute to the variation in microscopic susceptibility anisotropy imaging
**FIGURE S6** Microscopic frequency shift $\omega_{A,t_{0}}/\left(2\pi \right)$ estimated from a gradient‐echo scan with 3 head orientations at echo time *t* = 40.5 ms. For comparison, the second row shows the macroscopic frequency shift at standard head position before factoring out $B_{0}$‐direction dependence and the bottom section maps the DTI color‐encoded principal direction. The corpus callosum and cingulum bundle are primarily oriented perpendicular to the external magnetic field, whereas the fiber pathways traversing the posterior limb of the internal capsule run mostly parallel to it. These white matter regions show strong orientation‐dependent contrast in the frequency difference maps that is not evident in the microscopic frequency shift. Anterior corona radiata (ACR), anterior limb of internal capsule (ALIC), corpus callosum (CC), cingulum (CG), forceps major (FMAJ), forceps minor (FMIN), genu of corpus callosum (GCC), posterior corona radiata (PCR), posterior limb of internal capsule (PLIC), retrolenticular part of internal capsule (RLIC), splenium of corpus callosum (SCC), superior corona radiata (SCR), superior fronto‐occipital fasciculus (SFO), superior longitudinal fasciculus (SLF)^50^

## Data Availability

The data that support the findings of this study are available upon reasonable request. The software is openly available at https://ekaden.github.io.

## APPENDIX A

### A.1 SPHERICAL‐HARMONICS REPRESENTATION

For the estimation of the microscopic frequency shift $\omega_{A,t_{0}}\left(t\right)$, the microdomain population model (3) may alternatively be represented in spherical harmonic basis. Assuming that *p* is square‐integrable, Equation (3) can be reformulated as

(A1) $$\delta E_{t_{0}}\left({\hat{B}}_{0},\;t\right)=\sum_{\begin{array}{c} l=0,\\ l\;\text{even} \end{array}}^{N}\sum_{m=-l}^{l}\delta h_{l,t_{0}}\left(t\right)\;p_{l,m}Y_{l,m}\left({\hat{B}}_{0}\right)$$

up to finite order *N*, where $p_{l,m}$ denote the spherical harmonic coefficients of the axon orientation distribution *p* and $Y_{l,m}$ the real‐valued $L_{2}$‐normalized spherical harmonics. It is sufficient to consider the even indices *l* since *p* is antipodally symmetric. The Legendre coefficients of the microscopic gradient‐echo signal (2) take the form

(A2) $$\begin{array}{cc} \delta h_{l,t_{0}}\left(t\right)= & 2\pi \frac{\Gamma (l/2+1/2)}{\Gamma (l+3/2)}{\left(-i\omega_{A,t_{0}}\left(t\right)\left[t-t_{0}\right]\right)}^{l/2}\\ & \cdot_{1}F_{1}\left(l/2+1/2;l+3/2;-i\omega_{A,t_{0}}\left(t\right)\left[t-t_{0}\right]\right)\exp\left(i\omega_{A,t_{0}}\left(t\right)\left[t-t_{0}\right]\right) \end{array}$$

for even *l* = 0, 2, …, where Γ denotes the gamma function and ${\;}_{1}F_{1}$ the confluent hypergeometric function. We used the Funk–Hecke formula for the derivation of $\delta h_{l}$, noting that the microscopic gradient‐echo signal *δh* is antipodally symmetric and depends only on the angle $\left\langle {\hat{B}}_{0},\;u\right\rangle$ between the magnetic field direction ${\hat{B}}_{0}$ and the microdomain orientation *u*. Apart from that, model fitting follows Section 2.5.
