# Supplementary material for: Microscopic susceptibility anisotropy imaging
Source: Magn Reson Med. 2020 May 7;84(5):2739–53. doi: 10.1002/mrm.28303 (PMC7402021; doi:10.1002/mrm.28303)
Supplement: Supplementary file 1 — FIGURE S1 Maps of the microscopic frequency shift ωA,t0(t)/(2π), similar to Figure 4, obtained from gradient‐echo measurements with different numbers of magnetic field directions, but acquired with a 32‐channel phased‐array head coil offering a substantially higher signal‐to‐noise ratio at the expense of significantly less room for head movement that limits the achievable maximum angle between head orientations (here 39.2∘) FIGURE S2 Maps of the microscopic frequency shift ωA,t0(t)/(2π) estimated from a single gradient‐echo scan for different head orientations at echo time t = 40.5 ms. For comparison, the bottom row shows ωA,t0(t)/(2π) obtained from all 3 head positions relative to the main magnetic field. The 2 arrows point to white matter regions with fiber bundles mostly running parallel (left) and perpendicular to the external magnetic field in standard head orientation FIGURE S3 Differences of the microscopic frequency shift estimates from single head orientations with respect to a gradient‐echo experiment comprising all 3 head positions relative to the external magnetic field (bottom row) at echo time of 40.5 ms. Note that head orientation specific noise amplification effects, susceptibility‐induced image distortions, and residual spatial misalignment contribute to the variation in microscopic susceptibility anisotropy mapping FIGURE S4 Microscopic frequency shift ωA,t0(t)/(2π) obtained from gradient‐echo measurements at 2 head positions relative to the external magnetic field at echo time of 40.5 ms. For comparison, the bottom row shows the microscopic susceptibility anisotropy index estimated from all 3 head orientations. The 2 arrows indicate the pyramidal tract (left) and the superior longitudinal fasciculus, which are primarily oriented parallel and perpendicular to the main magnetic field in standard head position, respectively FIGURE S5 Differences of the microscopic frequency shift estimates from 2 head orientations with respect to a gradient‐echo meas [file MRM-84-2739-s001.pdf]

Supporting Information

Large-scale frequency variation removal

To eliminate residual long-range variation ψ in frequency difference measurements, such as the initial phase offset, we apply second-order 3D total generalised variation (TGV) regularisation [1]. Specifically, ψ is estimated using

$$\tilde{\psi} = \arg\min_{\psi} \frac{1}{2} \| My - M\psi \|_2^2 + \mathrm{TGV}_\alpha^2(\psi), \tag{S.1}$$

where y is the measured frequency difference, M is a mask operator that masks the image background and TGV_α^2 the second-order total generalised variation operator [1] with regularisation parameter α, which is chosen so that anatomical detail is preserved while spatially slowly varying frequency contributions are removed. To solve the convex optimisation problem given in Equation (S.1), we use a first-order primal-dual algorithm. Subsequently, the estimated large-scale frequency variation $\tilde{\psi}$ is subtracted from the measurements y to obtain, for each echo time t and magnetic field direction $\hat{B}_0$ separately, the frequency shift signal up to a global additive constant $\phi_{\hat{B}_0,t}$, which depends on the total image content.

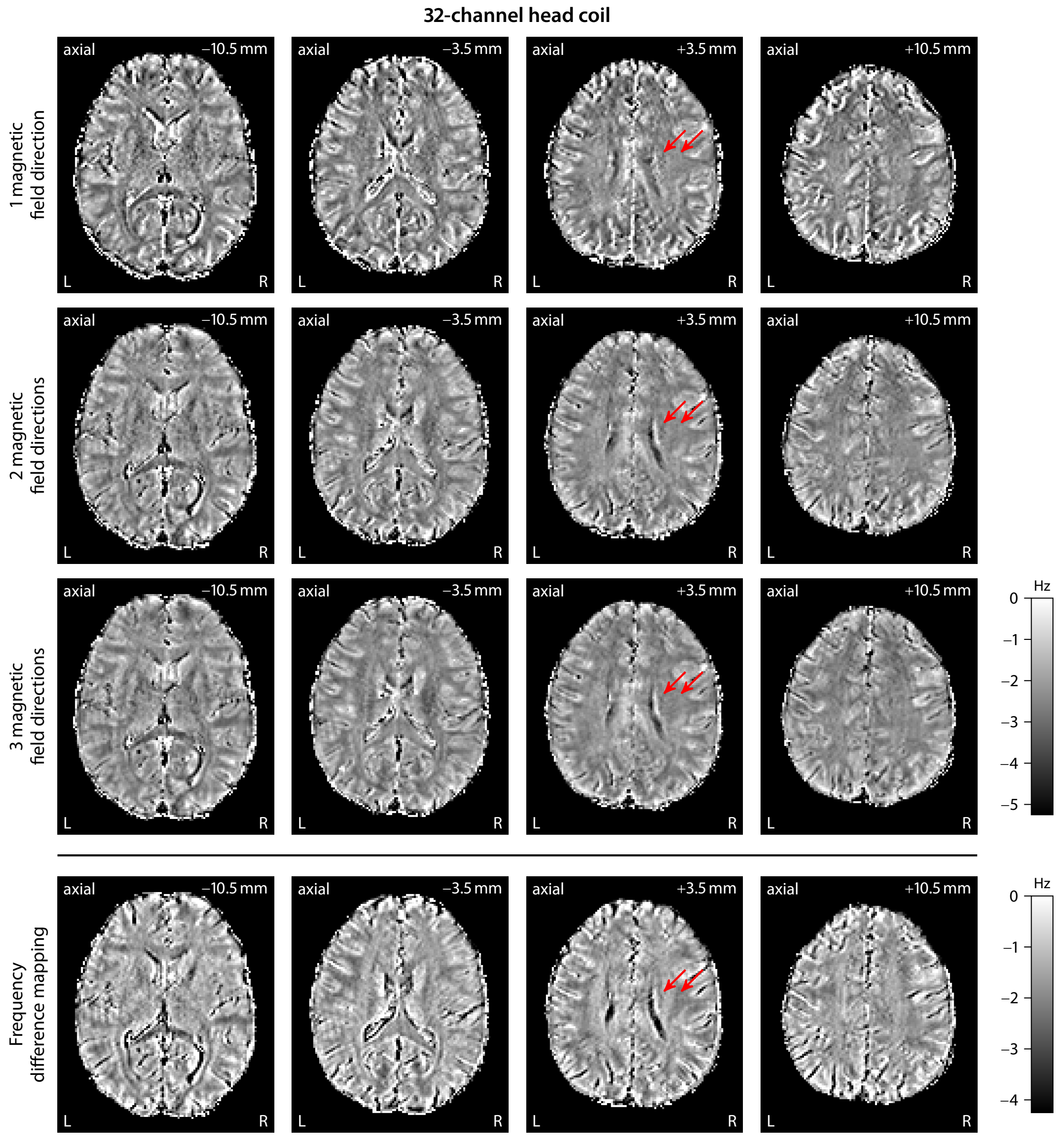

Figure S1: Maps of the microscopic frequency shift $\omega_{\mathrm{A},t_0}(t)/(2\pi)$, similar to Figure 4, obtained from gradient-echo measurements with different numbers of magnetic field directions, but acquired with a 32-channel phased-array head coil offering a substantially higher signal-to-noise ratio at the expense of significantly less room for head movement that limits the achievable maximum angle between head orientations (here 39.2°).

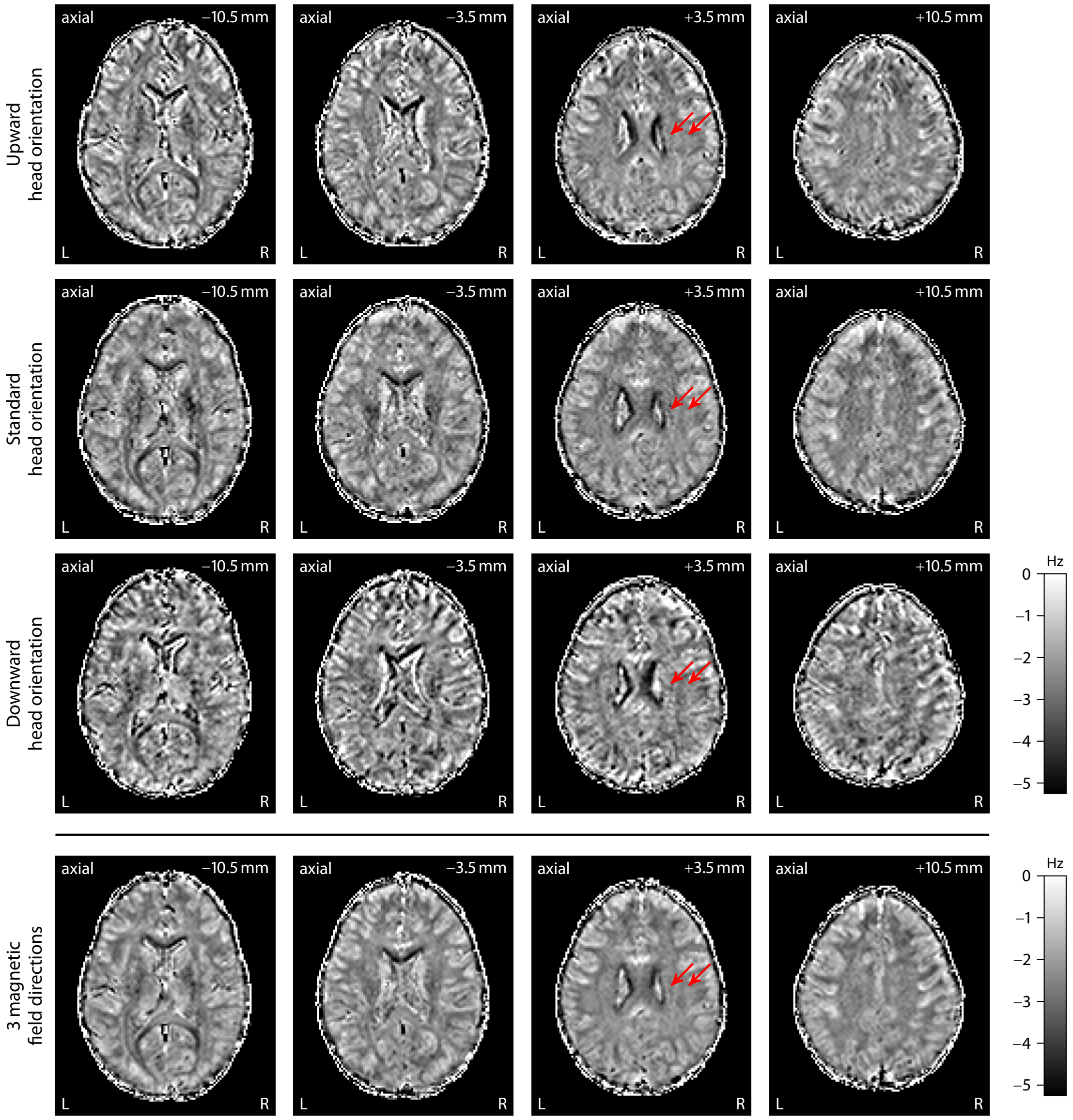

Figure S2: Maps of the microscopic frequency shift $\omega_{\mathrm{A},t_0}(t)/(2\pi)$ estimated from a single gradient-echo scan for different head orientations at echo time $t = 40.5$ ms. For comparison, the bottom row shows $\omega_{\mathrm{A},t_0}(t)/(2\pi)$ obtained from all three head positions relative to the main magnetic field. The two arrows point to white matter regions with fibre bundles mostly running parallel (left) and perpendicular to the external magnetic field in standard head orientation.

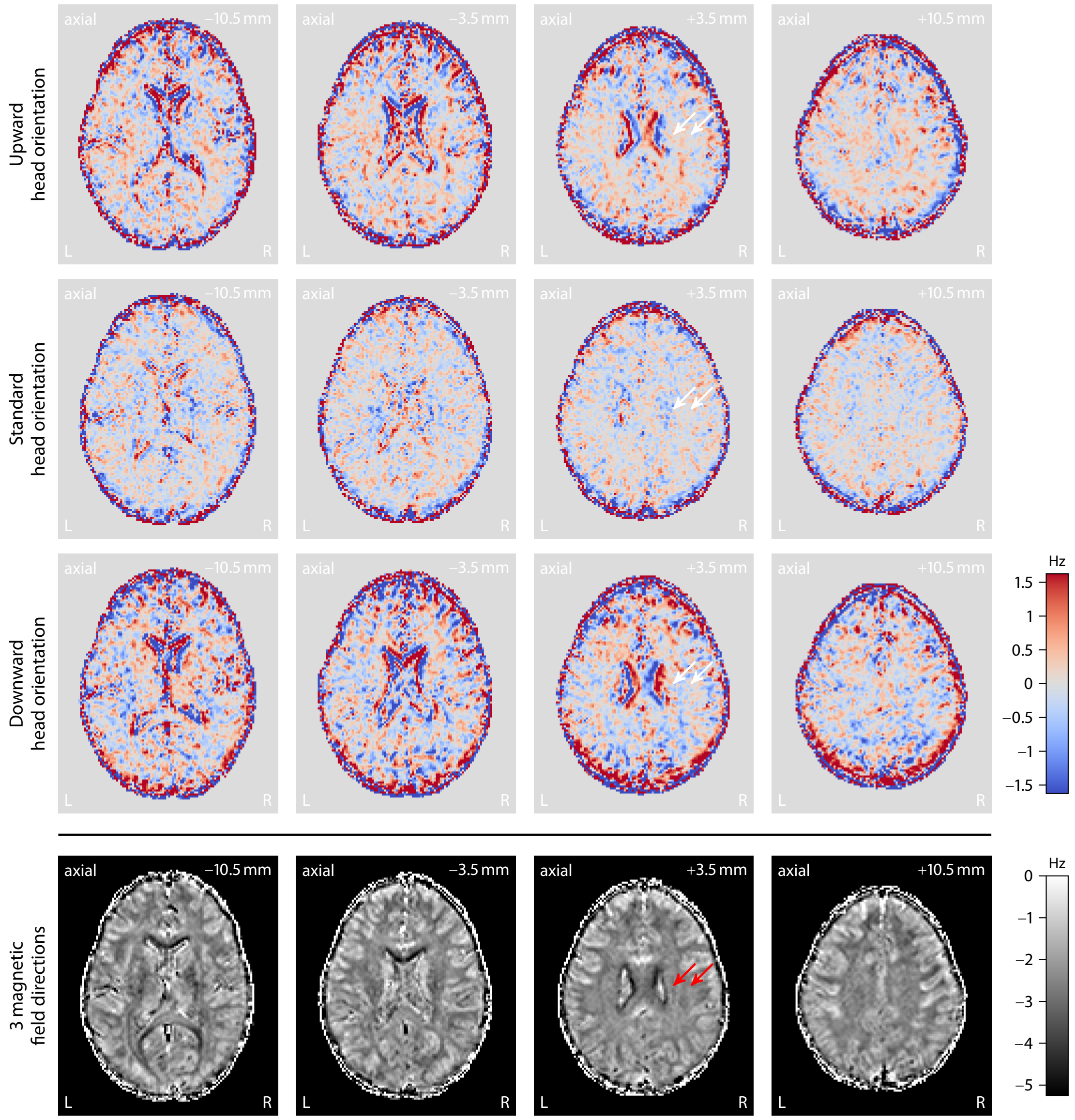

Figure S3: Differences of the microscopic frequency shift estimates from single head orientations with respect to a gradient-echo experiment comprising all three head positions relative to the external magnetic field (bottom row) at echo time of 40.5 ms. Note that head-orientation specific noise amplification effects, susceptibility-induced image distortions and residual spatial misalignment contribute to the variation in microscopic susceptibility anisotropy mapping.

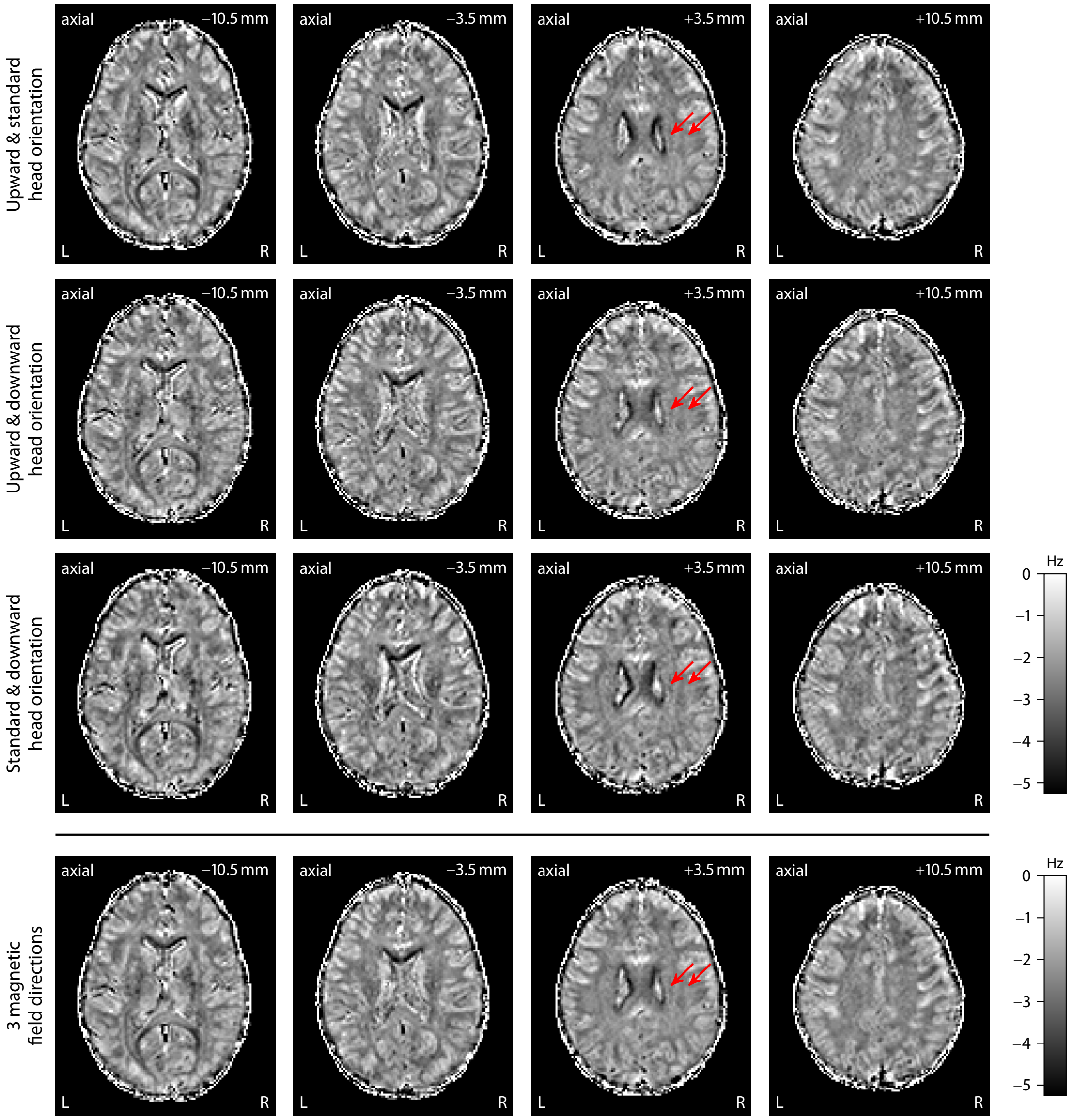

Figure S4: Microscopic frequency shift $\omega_{A,t_0}(t)/(2\pi)$ obtained from gradient-echo measurements at two head positions relative to the external magnetic field at echo time of 40.5 ms. For comparison, the bottom row shows the microscopic susceptibility anisotropy index estimated from all three head orientations. The two arrows indicate the pyramidal tract (left) and the superior longitudinal fasciculus, which are primarily oriented parallel and perpendicular to the main magnetic field in standard head position, respectively.

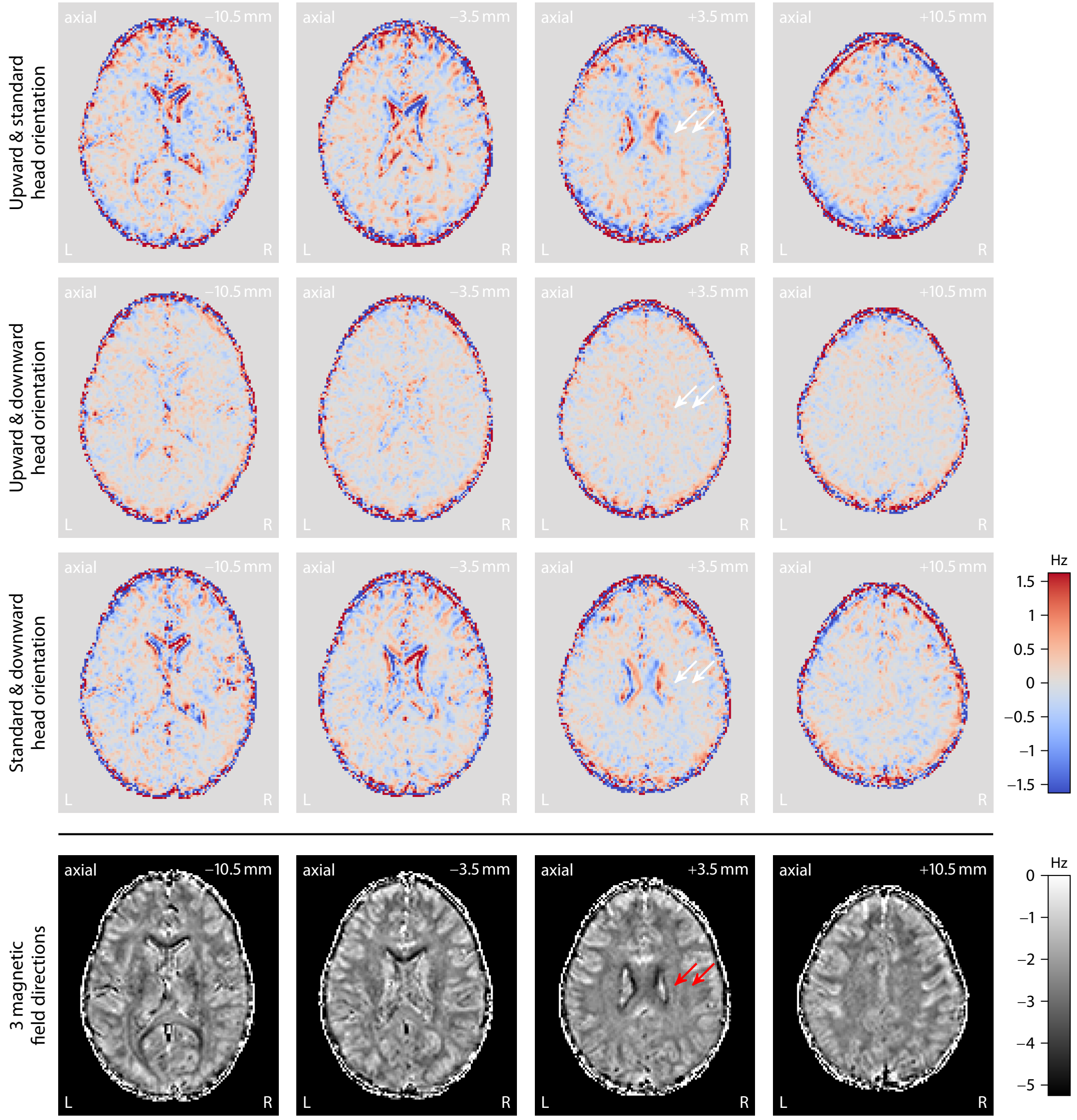

Figure S5: Differences of the microscopic frequency shift estimates from two head orientations with respect to a gradient-echo measurement comprising all three head positions relative to the external magnetic field (bottom row) at echo time $t = 40.5$ ms. Note that orientation-dependent noise amplification, susceptibility-induced distortions and residual spatial misalignment contribute to the variation in microscopic susceptibility anisotropy imaging.

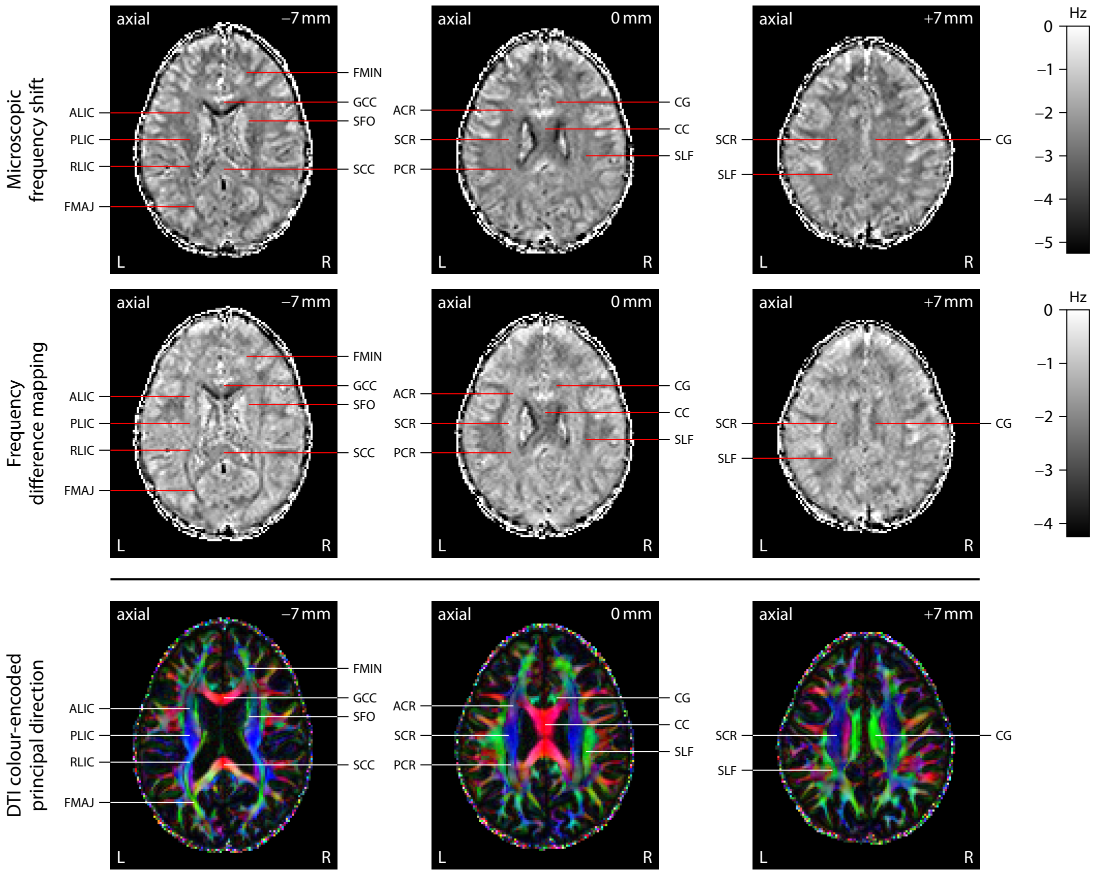

Figure S6: Microscopic frequency shift $\omega_{A,t_0}/(2\pi)$ estimated from a gradient-echo scan with three head orientations at echo time $t = 40.5$ ms. For comparison, the second row shows the macroscopic frequency shift at standard head position before factoring out B_0-direction dependence and the bottom section maps the DTI colour-encoded principal direction. The corpus callosum and cingulum bundle are primarily oriented perpendicular to the external magnetic field, whereas the fibre pathways traversing the posterior limb of the internal capsule run mostly parallel to it. These white matter regions show strong orientation-dependent contrast in the frequency difference maps that is not evident in the microscopic frequency shift. Abbreviations: anterior corona radiata (ACR), anterior limb of internal capsule (ALIC), corpus callosum (CC), cingulum (CG), forceps major (FMAJ), forceps minor (FMIN), genu of corpus callosum (GCC), posterior corona radiata (PCR), posterior limb of internal capsule (PLIC), retrolenticular part of internal capsule (RLIC), splenium of corpus callosum (SCC), superior corona radiata (SCR), superior fronto-occipital fasciculus (SFO), superior longitudinal fasciculus (SLF) [2].

References

1. Bredies K, Kunisch K, and Pock T. Total generalized variation. *SIAM Journal of Imaging Sciences*, 3: 492–526, 2010.

2. Mori S, Wakana S, Nagae-Poetscher LM, and van Zijl PCM. *MRI Atlas of Human White Matter*. Elsevier, 2005.
